# Supplementary material for: Isoquercitrin from Apocynum venetum L. Exerts Antiaging Effects on Yeasts via Stress Resistance Improvement and Mitophagy Induction through the Sch9/Rim15/Msn Signaling Pathway
Source: Antioxidants (Basel). 2023 Oct 31;12(11):1939. doi: 10.3390/antiox12111939 (PMC10669743; doi:10.3390/antiox12111939)
Supplement: Supplementary file 1 [file antioxidants-12-01939-s001.zip › antioxidants-2611540-supplementary.pdf]

Supplementary information

Isoquercitrin from *Apocynum venetum* L. Exerts Antiaging Effects on Yeasts via Stress Resistance Improvement and Mitophagy Induction through the Sch9/Rim15/Msn Signaling Pathway

Yanan Liu [1], Le Shen [1], Akira Matsuura [2], Lan Xiang [1,*] and Jianhua Qi [1,3,*]

[1] College of Pharmaceutical Sciences, Zhejiang University, Yu Hang Tang Road 866, Hangzhou 310058, China; liuyanan1231@zju.edu.cn (Y.L.); 3170101957@zju.edu.cn (L.S.).
[2] Department of Biology, Graduate School of Science, Chiba University, Chiba 263-8522, Japan; amatsuur@faculty.chiba-u.jp
[3] Jinhua Institute of Zhejiang University, Jinhua 321299, China
* Correspondence: lxiang@zju.edu.cn (L.X.); qijianhua@zju.edu.cn (J.Q.); Tel.: +86-0571-8820-8627 (J.Q.)

Supplementary Table S1. Yeast strains used in the present study

Strains	Genotype	Source
K6001	*MATa, ade2-1, trp1-1, can1-100, leu2-3,112, his3-11,15, GAL, psi+, ho::*HO*::CDC6* (at HO*), cdc6::hisG, ura3::URA3 GAL-ubiR-CDC6* (at *URA3)*	Gifted by Professor Michael Breitenbach
Δ*sod1*, Δ*sod2*, Δ*gpx*, Δ*cat*, Δ*uth1*, Δ*skn7*, Δ*atg2*, Δ*atg32* and Δ*rim15* of K6001	Replace the *SOD1* gene, *SOD2* gene, *GPx* gene, *CAT* gene, *UTH1* gen*e*, *SKN7* gene, *ATG2* gene, *ATG32* gene, and *Rim15* gene in K6001 with kanamycin gene, respectively	Constructed by Professor Akira Matsuura
YOM36	Prototrophic derivative of BY4742 (*MATα, his3Δ1, leu2Δ0, lys2Δ0, ura3Δ0*)	Gifted by Professor Akira Matsuura
BY4741	*MATa, his3Δ1, leu2Δ0, met15Δ0, ura3Δ0*	Gifted by Professor Akira Matsuura
YOM38 containing plasmid pRS316-*GFP-ATG8*	Prototrophic derivative of BY4742 (*MATα, his3Δ1, leu2Δ0, lys2Δ0*) containing plasmid pRS316-*GFP-ATG8*	Constructed by Professor Akira Matsuura
BY4741 with GFP fusion protein	*MATa, his3Δ1, leu2Δ0, met15Δ0, ura3Δ0* containing plasmid s*fGFP-Sch9-5HA::LEU2, Rim15-GFP::his3MX, Msn2-GFP::kanMX*	Constructed by Professor Akira Matsuura

Supplementary Table S2. The sequences of the primers for RT-PCR analysis

Primers	Sequences
MSN2, sense	5′-AGA ACG ATA TGC TGC CGA ATT C-3′
antisense	5′-CGC CAC TTT CGC AAT AAC G-3′
MSN4, sense	5′-GGA TTG ATG GAC CCG GTA TTG-3′
antisense	5′-CCA AAG GTA TAT TCC GGC GAA-3′
TUB1, sense	5′-CCA AGG GCT ATT TAC GTG GA-3′
antisense	5′-GGT GTA ATG GCC TCT TGC AT-3′

Supplementary Table S3. Replicative lifespan of K6001 yeasts and its mutants

Figures	Yeast strains	Treatment (μM)	Mean lifespan (generations)
Fig.5a, 5b	K6001	Control	6.83 ± 0.46
		RES-10	8.95 ± 0.56**
		IQ-30	10.25 ± 0.61***
	Δ*sod1* of K6001	Control	6.08 ± 0.36
		RES-10	6.90 ± 0.36
		IQ-30	7.13 ± 0.41
	Δ*sod2* of K6001	Control	6.40 ± 0.36
		RES-10	7.03 ± 0.34
		IQ-30	7.43 ± 0.39
Fig.5c, 5d	K6001	Control	6.28 ± 0.44
		RES-10	9.30 ± 0.58***
		IQ-30	10.48 ± 0.69***
	Δ*cat* of K6001	Control	6.00 ± 0.37
		RES-10	6.78 ± 0.40
		IQ-30	7.43 ± 0.46
	Δ*gpx* of K6001	Control	6.00 ± 0.30
		RES-10	6.65 ± 0.37
		IQ-30	7.25 ± 0.48
Fig.5e, 5f	K6001	Control	6.78 ± 0.41
		RES-10	9.30 ± 0.63**
		IQ-30	9.00 ± 0.54**
	Δ*skn7* of K6001	Control	7.70 ± 0.39
		RES-10	7.18 ± 0.43
		IQ-30	7.40 ± 0.48
	Δ*uth1* of K6001	Control	8.88 ± 0.62#
		RES-10	6.08 ± 0.36
		IQ-30	6.70 ± 0.40
Fig.6g, 6h	K6001	Control	7.05 ± 0.46
		RES-10	11.05 ± 0.56***
		IQ-30	10.30 ± 0.56***
	Δ*atg2* of K6001	Control	6.03 ± 0.33
		RES-10	7.13 ± 0.50
		IQ-30	6.05 ± 0.31
	Δ*atg32* of K6001	Control	6.33 ± 0.37
		RES-10	6.25 ± 0.37
		IQ-30	5.58 ± 0.27
Fig.6i	K6001	Control	7.45 ± 0.34
		GSK3-IN-3-1	8.84 ± 0.43*
		GSK3-IN-3-3	10.70 ± 0.47***
		GSK3-IN-3-10	9.65 ± 0.42**
		Mdivi-1-0.3	8.61 ± 0.41
		Mdivi-1-1	8.49 ± 0.40
		Mdivi-1-3	8.41 ± 0.36
Fig.7c	K6001	Control	6.70 ± 0.37
		RES-10	10.05 ± 0.59***
		IQ-10	10.23 ± 0.63***
	Δ*rim15* of K6001	Control	6.63 ± 0.38
		RES-10	7.88 ± 0.48#
		IQ-10	8.03 ± 0.52#

*, ** and *** represent significant differences compared to the control group of K6001 at $p < 0.05$, $p < 0.01$ and $p < 0.001$, respectively. # represents significant differences compared to the control group of Δ*rim15* of K6001 at $p < 0.05$.

2. Materials and Methods

2.1 The methods of lifespan assay

In short, in the replicative lifespan assay, the K6001 yeast was transferred into a 15 mL centrifuge tube after thawing at 37 °C, washed three times with 5 mL of phosphate buffer solution (PBS), divided into tubes containing approximately 5 mL of galactose liquid medium (3% galactose, 2% hipolypeptone, and 1% yeast extract), and cultured for 12-24 h with shaking (180 rpm, 28 °C). On day 2, the yeasts that reached the logarithmic growth phase were washed three times and diluted with PBS, and approximately 4000 cells were spread on yeast extract peptone dextrose (YPD) agar plates (2% glucose, 2% hipolypeptone, 1% yeast extract, and 2% agar) containing resveratrol, IQ, GSK3-IN-3 and Mdivi-1 at the test concentration. The agar plates were cultured for 48 h at 28 °C. Forty microcolonies that formed on each agar plate were randomly selected for observation under the microscope, and daughter cells in the range of 4–20 produced by one mother cell were counted. The replicative lifespan assay of the Δ*sod1,* Δ*sod2,* Δ*uth1,* Δ*skn7,* Δ*gpx,* Δ*cat,* Δ*atg2,* Δ*atg32,* and Δ*rim15* yeast strains with K6001 background was similar to that of the K6001 yeast.

In the chronological lifespan assay, YOM36 yeasts were cultured in YPD medium (2% glucose, 2% hipolypeptone, and 1% yeast extract) in a shaking incubator (180 rpm, 28 °C). On day 2, the culture medium was replaced with synthetic defined (SD) medium (0.17% yeast nitrogen base without amino acids and ammonium sulphate, 0.5% ammonium sulphate, and 2% glucose). After 24 h of cultivation, the yeast suspension was added in 100 mL of SD medium containing IQ at concentrations of 0, 1, 10, and 30 μM with an initial optical density at 600 nm (OD_{600}) of 0.01 (defined as day 0). Rapamycin at 1 μM was applied as positive control. At day 3, a total of 200 yeasts were spread on a YPD agar plate, and the colony-forming units (CFUs) on the plates were counted after 48 h of incubation. This step was carried out every 2 days until the survival rate was below 5%, and the CFUs at the third day were considered 100% survival.

2.2 Measurement of the Concentration of Proteins

BCA assay kit (CoWin Biotech, Beijing, China) was used for measuring the concentration of proteins in the supernatant which was obtained from yeast lysates after centrifugation. Briefly, 200 μL of BCA working solution (A: B = 50: 1) and 25 μL of each gradient diluted standard protein or samples were added into one well of 96-well plate, and each sample was repeated two times. Subsequently, the 96-well plate was incubated at 37 °C for 25 min, and the absorbance of samples at 562 nm was measured by using a BioTek Microplate Reader (BioTek, Winooski, USA).

2.3 Measurement of RNS, MDA Levels and Sod, Cat, Gpx Enzymatic Activity on Yeast Cells

For RNS assay, firstly, dilute the O_{52} probe 10 times with pure water according to the number of samples. Add 198 μL protein sample to the black 96-well plate, and then add 2 μL diluted O_{52} probe, mix them well. Then the plate was incubated in dark at 37 °C for 30 minutes. The fluorescence intensity of sample was measured with excitation and emission wavelengths of 488 and 530 nm by using a Spark micro-plate reader (Tecan Trading Co., Ltd, Männedorf, Switzerland).

For MDA assay, briefly, 100 μL of ethanol, 10 nmol/mL standard or test samples were added to 1.5 mL Eppendorf tubes respectively. The tubes were vortexed well after adding 100 μL reagent I. Subsequently, 375 μL reagent II and 125 μL reagent III were added into the tubes in turn. Finally, these tubes were sealed and heated in a water bath for 80 min at 95 °C. Then, 200 μL of supernatant was taken into each well of a 96-well plate after centrifugation (3500–4000 ×g/min, 10 min), and the absorbance at 532 nm was measured using a BioTek microplate reader (BioTek, Winooski, USA). MDA content in

yeast (nmol/mg protein) = (determination group OD value / [standard group OD value−blank group OD value]) × standard concentration (10 nmol/mL) ÷ protein concentration of sample (mg protein/mL).

For Sod enzyme activity assay, 25 μg protein in each group was first mixed with reagent VII and vortexed for 1 min to inactivate the Mn-Sod enzyme activity in the samples. The supernatant was obtained for detecting the CuZn-Sod enzyme activity after centrifugation (3500×g, 15 min). The reagent I, blank control, samples, and the samples treated by reagent VII were added to the 96-well plate according to the dosage in the manufacturer instructions. Then, reagents II, III, and IV were added into each well. Then, the plate was incubated at 37 °C for 40 min after mixing well. Finally, the A550 absorbance value of samples was measured after reacting with 120 μL of chromogenic working fluid at room temperature for 10 min under dark. Activity of Sod enzyme = ([control group OD value - determination group OD value] / control group OD value) / 50% × (total volume of reaction solution / sample volume)/ protein concentration of sample.

During Cat enzyme activity assay, gradient concentrations of hydrogen peroxide solution were first prepared. Afterward, chromogenic working fluid was added to the 96-well plate to mix with hydrogen peroxide solution and reacted at 25 °C for 15 min. The standard curve of hydrogen peroxide concentration was determined after measuring the absorption value at 520 nm. Simultaneously, catalase buffer and 250 mM of hydrogen peroxide were added to each well with 5 μL protein samples (1.25 μg/μL). After reacting at 25 °C for 1–5 min, 450 μL of enzyme reaction termination solution was added to terminate the reaction. Then, 10 μL mixture was taken to react with chromogenic working fluid at 25 °C for 15 min, and the absorption value at 520 nm was measured. Sample catalase activity = [consumption of micromole of hydrogen peroxide] × [dilution ratio] / ([reaction minutes] × [sample volume] × [protein concentration]), and [consumed micromole of hydrogen peroxide] = [micromole of residual hydrogen peroxide in blank control] − [micromole of residual hydrogen peroxide of sample].

For Gpx enzyme activity assay, 12.5 μg protein of each sample was taken. The general process is that the Gpx detection buffer, samples, Gpx detection working solution, and peroxide reagent were added in a 96-well plate in turn. The absorbance value of A340 was measured every 3 min and for six times after mixing well. The activity of Gpx in the detection system = [(ΔA340 (sample) − ΔA340 (blank))/min] / (0.00622 $\mu M^{-1} cm^{-1}$ × 0.276 cm). Total Gpx activity in the sample = Gpx activity in the detection system × dilution ratio / sample protein concentration.

2.4 Real-Time Polymerase Chain Reaction (RT-PCR) Analysis

The BY4741 yeasts were cultured in 20 mL YPD containing isoquercitrin at 0, 1, 10, and 30 μM or RES at 10 μM for corresponding time, respectively. After the co-incubation, the yeasts in each group were harvested and washed three times with PBS. Then the total RNA was extracted by grinding at 68 Hz for 3 min after adding 1 mL TRIzon reagent (CoWin Biotech, Beijing, China) and appropriate amount of grinding beads. A total of 200 μL of chloroform was added and mixed, then the colorless water layer was obtained after centrifugation (4 °C, 12,000 rpm, 15 min). The isopropanol with the same volume as colorless water layer was added to precipitate the total RNA. The total RNA was harvested after centrifugation (4 °C, 12,000 rpm, 10 min) and washed with 75% ethanol, then dissolved in RNase-free water (CoWin Biotech, Beijing, China). The RNA concentration was determined by using an Eppendorf Biophotometer Plus (Eppendorf Company, Hamburg, Germany). Reverse transcription was performed using 5 μg of total RNA and a HiFi-MMLV cDNA Kit (CoWin Biotech, Beijing, China). Quantitative RT-PCR was conducted by using a CFX96 Touch (BioRad, Hercules, USA) and SYBR Premix EX Taq ™ (Takara, Otsu, Japan).

Supplementary Figures

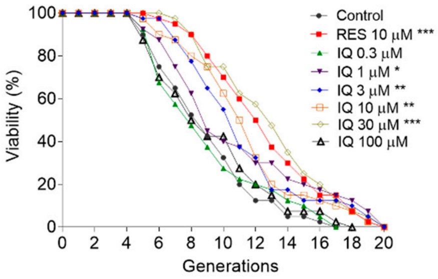

Supplementary Figure S1. Effect of IQ on the replicative lifespan of K6001 yeasts. The average lifetime of each group was as follows: control (8.05 ± 0.47), RES at a dose of 10 μM (11.63 ± 0.55), IQ at a dose of 0.3 μM (8.18 ± 0.57), IQ at a dose of 1 μM (9.88 ± 0.73), IQ at a dose of 3 μM (10.18 ± 0.60), IQ at a dose of 10 μM (10.55 ± 0.57), IQ at a dose of 30 μM (11.93 ± 0.60), IQ at a dose of 100 μM (8.18 ± 0.54). *, ** and *** represent significant differences compared to the control group at $p < 0.05$, $p < 0.01$ and $p < 0.001$, respectively.

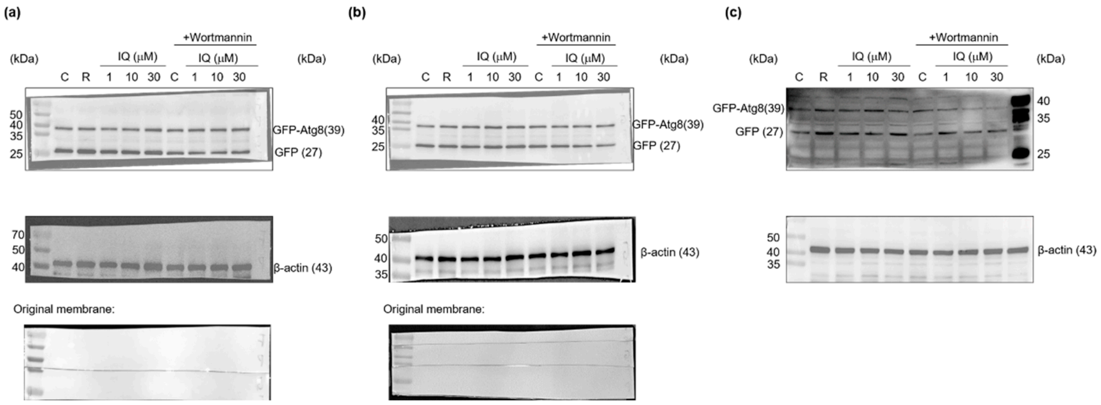

Supplementary Figure S2. The full unedited gel for western blot analysis of GFP-Atg8, free GFP, and β-actin in yeast in Figure 6d. (a) (b) The GFP-Atg8, free GFP and β-actin are obtained from one membrane by using stripping buffer; (c) The GFP-Atg8, free GFP and β-actin are obtained from two membranes by loading same amount of protein**.** Protein bands are obtained by exposure via Bio-Rad chemiluminescence imager (Bio-Rad Laboratories, Hercules, California, USA).

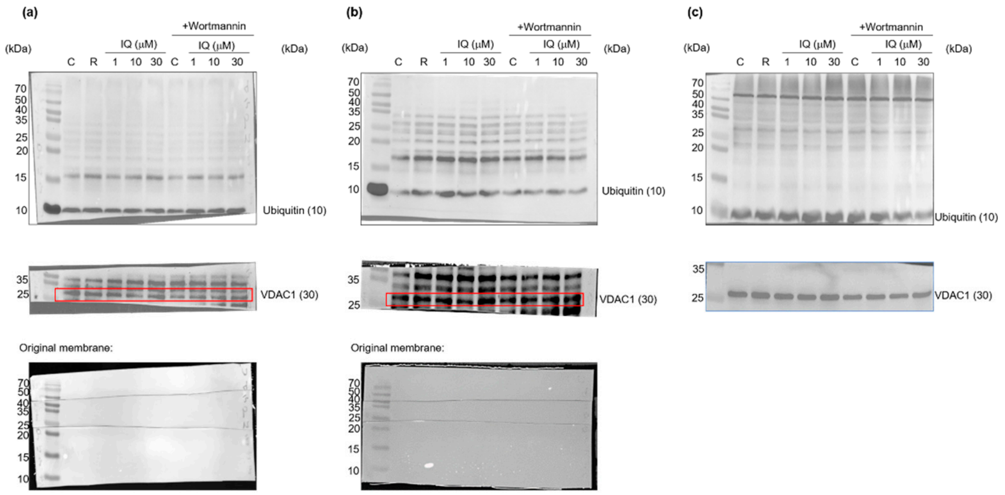

Supplementary Figure S3. The full unedited gel for western blot analysis of ubiquitin and VDAC1 in yeast in Figure 6f. (a) (b) The ubiquitin and VDAC1 are obtained from one membrane by using stripping buffer; (c) The ubiquitin and VDAC1 are obtained from two membranes by loading same amount of protein. Protein bands are obtained by exposure via Bio-Rad chemiluminescence imager (Bio-Rad Laboratories, Hercules, California, USA).

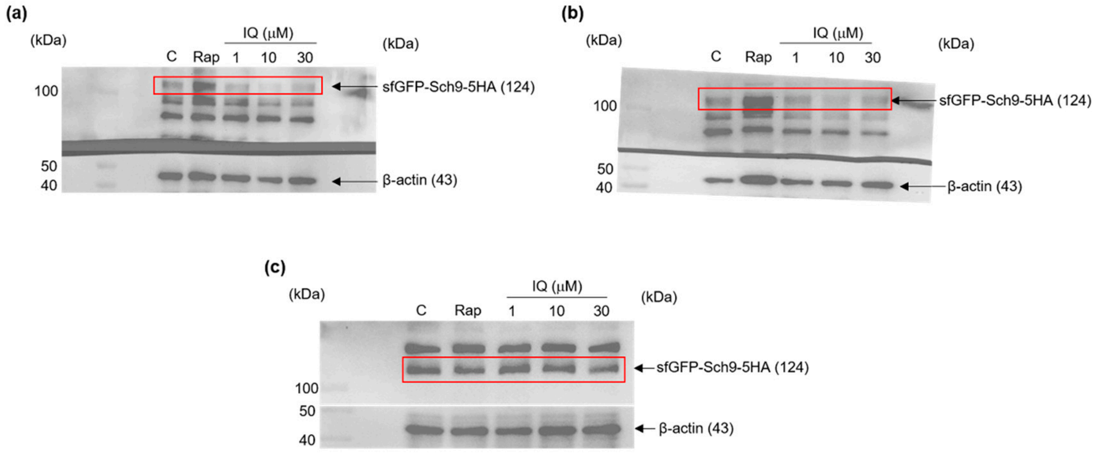

Supplementary Figure S4. The full unedited gel for western blot analysis of sfGFP-Sch9-5HA and β-actin of yeast in Figure 7a. (a) (b) The sfGFP-Sch9-5HA and β-actin are obtained from one membrane; (c) The sfGFP-Sch9-5HA and β-actin are obtained from two membranes by loading same amount of protein. Protein bands are obtained by exposure via Bio-Rad chemiluminescence imager (Bio-Rad Laboratories, Hercules, California, USA).

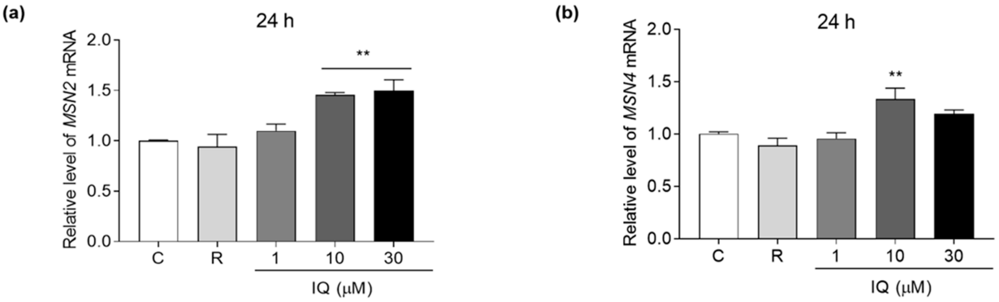

Supplementary Figure S5. Effects of IQ on *MSN2* and *MSN4* genes expression in yeasts. (a) The genes expression of *MSN2* in yeast after treatment with isoquercitrin for 24 h. (b) The genes expression of *MSN4* in yeast after treatment with isoquercitrin for 24 h. Experiments were repeated thrice, and the data are presented as mean ± SEM. ** indicates significant differences from the control group at $p < 0.01$.
